# Supplementary material for: The genome sequence of the harbour porpoise, Phocoena phocoena (Linnaeus, 1758)
Source: Wellcome Open Res. 2025 Apr 8;10:181. [Version 1] doi: 10.12688/wellcomeopenres.24011.1 (PMC12006785; doi:10.12688/wellcomeopenres.24011.1)
Supplement: Peer review history

# Reviewer report: Reviewer response for version 1

**DOI:** 10.21956/wellcomeopenres.26491.r122161

**Published:** 2025-04-17

**Authors:** Mariana F. Nery

**Affiliations:** 1 Universidade Estadual de Campinas, Campinas, State of São Paulo, Brazil

The manuscript presents a high-quality, chromosome-level genome assembly for the harbour porpoise (Phocoena phocoena), an odontocete cetacean broadly distributed in temperate coastal waters of the Northern Hemisphere. The assembly is highly contiguous and complete, with 94.41% of the sequence assigned to 22 chromosomal pseudomolecules. The authors employed PacBio HiFi long-read sequencing combined with Hi-C data, resulting in excellent quality metrics.

 The methodology is robust and well-documented, including protocols for high molecular weight DNA and RNA extraction, library preparation, and genome assembly. The genome meets or exceeds current standards established by the Earth BioGenome Project. 

 This genome assembly represents a valuable resource for marine mammal genomics and conservation biology. It is well-executed, well-described, and openly accessible. 

 Minor point: while RNA-Seq data is mentioned, its role in gene annotation or transcriptome analysis is not discussed.

Are sufficient details of methods and materials provided to allow replication by others?

Yes

Is the rationale for creating the dataset(s) clearly described?

Yes

Are the datasets clearly presented in a useable and accessible format?

Yes

Are the protocols appropriate and is the work technically sound?

Yes

Reviewer Expertise:

Genomics, molecular evolution

I confirm that I have read this submission and believe that I have an appropriate level of expertise to confirm that it is of an acceptable scientific standard.

# Reviewer report: Reviewer response for version 1

**DOI:** 10.21956/wellcomeopenres.26491.r122159

**Published:** 2025-04-17

**Authors:** Takushi Kishida

**Affiliations:** 1 Nihon University, Kanagawa, Japan

This study provides a high-quality chromosomal-level harbor porpoise haploid genome assembly. I have no complaints about the quality of this genome data.

 I hope, if possible, the authors deposit the specimen used for genome sequencing in an appropriate museum so that a vouchered specimen associated with this genome data is available.

Are sufficient details of methods and materials provided to allow replication by others?

Yes

Is the rationale for creating the dataset(s) clearly described?

Yes

Are the datasets clearly presented in a useable and accessible format?

Yes

Are the protocols appropriate and is the work technically sound?

Yes

Reviewer Expertise:

Evolutionary genomics, population genetics

I confirm that I have read this submission and believe that I have an appropriate level of expertise to confirm that it is of an acceptable scientific standard.
